# The Final (Oral Ebola) Vaccine Trial on Captive Chimpanzees?

**DOI:** 10.1038/srep43339

**Published:** 2017-03-09

**Authors:** Peter D. Walsh, Drishya Kurup, Dana L. Hasselschwert, Christoph Wirblich, Jason E. Goetzmann, Matthias J. Schnell

**Affiliations:** 1 Department of Archaeology and Anthropology, University of Cambridge, Cambridge CB2 3QG, UK; 2 Department of Microbiology and Immunology, Sidney Kimmel Medical College, Thomas Jefferson University, Philadelphia, PA, 19438, USA; 3 University of Louisiana Lafayette, New Iberia Research Center, New Iberia, LA, 70560, USA

## Abstract

Could new oral vaccine technologies protect endangered wildlife against a rising tide of infectious disease? We used captive chimpanzees to test oral delivery of a rabies virus (RABV) vectored vaccine against Ebola virus (EBOV), a major threat to wild chimpanzees and gorillas. EBOV GP and RABV GP-specific antibody titers increased exponentially during the trial, with rates of increase for six orally vaccinated chimpanzees very similar to four intramuscularly vaccinated controls. Chimpanzee sera also showed robust neutralizing activity against RABV and pseudo-typed EBOV. Vaccination did not induce serious health complications. Blood chemistry, hematologic, and body mass correlates of psychological stress suggested that, although sedation induced acute stress, experimental housing conditions did not induce traumatic levels of chronic stress. Acute behavioral and physiological responses to sedation were strongly correlated with immune responses to vaccination. These results suggest that oral vaccination holds great promise as a tool for the conservation of apes and other endangered tropical wildlife. They also imply that vaccine and drug trials on other captive species need to better account for the effects of stress on immune response.

In 2014 the world was gripped by fears of an Ebola virus (EBOV) pandemic. Few people realized that Ebola had already inflicted pandemic scale mortality on our closest relatives, killing about one third of the world’s gorillas and countless chimpanzees (Fig. 1)^1^,^2^. African apes are also threatened by naturally occurring pathogens like anthrax, SIV (the wild ape progenitor of HIV), and the increasing spillover of human pathogens, not to mention rampant commercial poaching and habitat loss^3^. A glimmer of hope lies in the fact that many of the disease threats are vaccine preventable. Unfortunately, traditional hypodermic dart delivery of vaccine is extremely challenging for animals that live at low density in thick tropical forest and fear humans. A solution to this problem is suggested by the use of oral vaccines to virtually eradicate of fox rabies from Western Europe^4^. Despite the astoundingly good safety record of live virus (replication competent) vaccines in the oral rabies program, vocal opposition both to the release of genetically modified organisms (i.e. recombinant vaccines) and to the use of replication competent vaccines^5^ has limited oral vaccination of endangered wildlife to a single study published in 2016: the successful test of an oral rabies vaccine on *Critically Endangered* Ethiopian wolves^6^.

By orally vaccinating charismatic apes threatened by the scariest of emergent pathogens we hoped to focus further attention on the extent to which conservation concerns may balance fears about vaccine safety: to push the debate on orally vaccinating endangered wildlife from an entrenched ideological deadlock towards a scientific evidence-based discussion of measurable costs and benefits. Our first step was to conduct trials on oral delivery of an EBOV vaccine to captive chimpanzees. We started with a captive trial because host evolutionary similarity is strongly correlated with disease susceptibility and vaccine effectiveness^7^: an effect implied in the fact that several early vaccines that were protective against Ebola challenge in mice were not protective in macaques^8^. Thus, management authorities in Africa had scientific justification for insisting that before an EBOV vaccine was used on endangered apes in the wild it should be first tested on captive apes.

Here we report results from a trial of the filorab1 vaccine, which inserts the gene encoding the EBOV glycoprotein (GP) into the replication competent but highly attenuated SAD B19-based RABV vaccine^9^.We vaccinated ten chimpanzees at the University of Louisiana Lafayette’s New Iberia Research Center with 1.5 × 10^8^ focus forming units of vaccine, six orally and four intramuscularly (IM). We chose filorab1 both because it confers robust protection to macaques vaccinated IM then challenged with EBOV and because the high safety and effectiveness of the parent rabies vaccine has been exhaustively documented in extensive captive trials on a variety of mammals and through distribution of millions of vaccine-laced oral baits in Western Europe^10^.

The first goal of the trial was to evaluate whether the offspring filorab1 vaccine was immunogenic when delivered orally to chimpanzees. We did not challenge with EBOV but simply monitored post-vaccination immune response by assaying blood drawn on days -8, 0, 7, 14 and 28. New Endangered Species Act (ESA) regulations banning invasive research on chimpanzees forced termination of the trial after Day 28. To evaluate immunogenicity we performed enzyme-linked immunosorbant assays (ELISA) of antibodies specific to EBOV GP and RABV as well as assays of serum neutralizing activity against RABV and EBOV GP pseudo-typed vesicular stomatitis virus (VSV-EBOV).

The second goal of the trial was to evaluate the health impact of the vaccine trial. To quantify the impact of the vaccine itself we monitored body mass and a standard panel of hematology and blood chemistry parameters (raw data are available in Supplementary Tables S1,S2,S3). The hematology and blood chemistry data included several variables whose values show well-documented correlations with psychological stress, including white blood cell count (WBC) and serum glucose. Our interest in psychological stress was twofold. First, in advocating a ban on the use of chimpanzees in biomedical research, animal welfare advocates argued that

“All invasive research is torture. And it’s not just the procedures. It’s the imprisonment. It’s being kept in a small space with no choice”^11^.

We sought both to objectively quantify the level of stress experienced by study chimpanzees and to differentiate between chronic stress induced by social isolation or confinement in small experimental cages and acute stress induced by the vaccine or experimental procedures (e.g. sedation or changes in housing). Second, research on humans has shown that acute psychological stress can have strong effects on immune function^12^, most notably a stimulatory effect on post-vaccination antibody production^13^. Although a large literature documents strong stress responses of chimpanzees and other captive animals to experimental conditions we could find little published work documenting or controlling for the effects of such stressors on captive animal immune response during vaccine trials.

## Results

**Figure 2:**
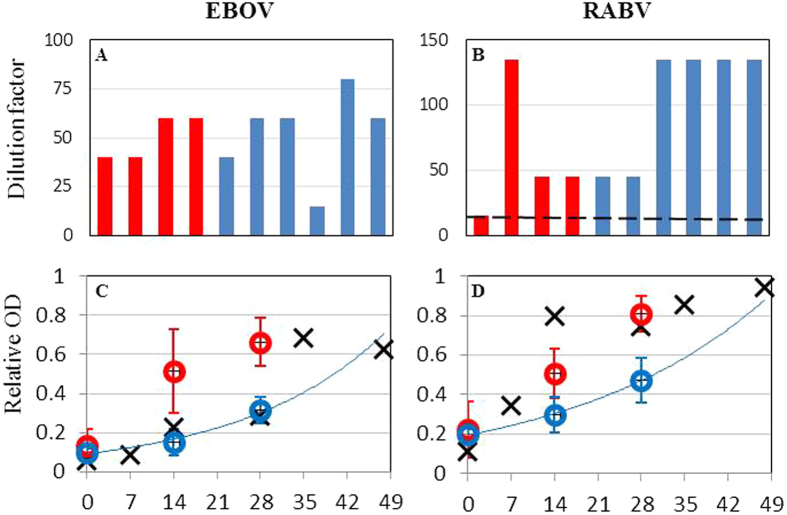
Filorab1 vaccine is strongly immunogenic in chimpanzees. Top Panels: Highest serum dilution factor at which serum antibodies isolated from chimpanzees achieved 50% neutralization of (**A**) EBOV pseudotyped VSV and (**B**) RABV. Red bars IM vaccinated chimpanzees, blue bars orally vaccinated chimpanzees. Serum antibodies from all subjects except one IM vaccinated chimpanzee achieved 50% RABV neutralization at dilutions much higher than the lowest dilution factor (dashed line in (**B)** considered by the World Health Organization to be robustly protective against RABV challenge. No comparable standard is accepted for EBOV. Bottom Panels: ELISA optical densities (OD) for chimpanzee serum titers of IgG against (**C**) EBOV GP and **D**) RABV. Day 0, 14, and 28 OD’s for 1/150 dilutions of chimpanzee IgG are plotted as a proportion of the OD for the positive control: post-challenge IgG from macaques vaccinated with filorab1 in a previous study^14^. Circles are averages for the six orally vaccinated (in blue) and four IM vaccinated (in red) chimpanzees. Least squares regression lines through the orally vaccinated chimpanzee data show very close to exponential growth of IgG against EBOV GP IgG (R^2^ = 0.98) and RABV IgG (R^2^ = 0.99). Error bars are 95% confidence intervals (1.96 standard errors). Lack of confidence interval overlap between successive sampling days indicates highly significant rises in IgG on Days 14 and 28. RABV IgG titers for orally vaccinated chimpanzees grew more slowly than for IM vaccinated chimpanzees or macaques in the previous study (black X’s). EBOV GP IgG titers of orally vaccinated chimpanzees grew at a rate similar to that of macaques.

Vaccination with filorab1 provoked robust immune responses. EBOV GP-specific antibodies isolated from Day 28 serum of both oral and IM chimpanzees achieved 50% neutralization of VSV-EBOV at a dilution factor comparable to that Day 28 serum Rhesus macaques who, in a previous study, were IM vaccinated with filorab1 then survived EBOV challenge (Fig. 2A)^14^. Chimpanzee serum antibodies also achieved robust neutralization of RABV, with all animals showing anti-rabies activity greater than that considered by the World Health Organization to be protective against RABV infection (Fig. 2B). For both EBOV and RABV, oral and IM chimpanzees did not differ significantly in the maximum dilution at which 50% neutralization was achieved.

ELISA also indicated robust immune responses. In orally vaccinated chimpanzees serum EBOV GP-specific immunoglobulin G (EBOV GP-IgG) (Fig. 2C) increased at a rate very close to exponential (Fig. 2), reaching a Day 28 mean slightly higher than the Day 28 value seen in IM vaccinated Rhesus macaques in the previous study. IM vaccinated chimpanzees started the study with slightly higher EBOV GP-IgG than orally vaccinated chimpanzees or macaques and reached a Day 28 peak comparable to the Day 35 peak seen in the longer (45 day) macaque study. The rate of increase in the EBOV GP-IgG titers of oral chimpanzees accelerated slightly during the trial (exponential increase rate Day 0–14 = 0.033, Day 14–28 = 0.052). This later “kick in” of antibody increases in orally vaccinated chimpanzees is consistent with the smaller vaccine inoculum introduced into the bloodstream through oral vaccination. Projecting the Day 0–28 exponential increase rate forward (Fig. 2C) suggests that oral chimpanzees would have reached the peak titers seen in macaques on Day 57 (or Day 51 using the Day 14–28 increase rate). We could not verify these projections because imposition of the new ESA regulations truncated the study.

Vaccinated chimpanzee sera also exhibited robust rises in RABV-specific IgG (r-IgG) (Fig. 2D). IM chimpanzees showed Day 28 r-IgG titers slightly higher than Rhesus macaques vaccinated with filorab1 in the previous study. Orally vaccinated chimpanzees again showed lower Day 28 antibody titers than IM chimpanzees and macaques but consistent exponential increase during the study (exponential increase rate Day 0–14 = 0.030, Day 14–28 = 0.033). Day 28 r-IgG and EBOV-IgG were also highly correlated (Pearson correlation R^2^ = 0.9, p = 0.00003): likely reflecting the total dependence of EBOV antigen growth dynamics on filorab1 (RABV) replication.

**Figure 3:**
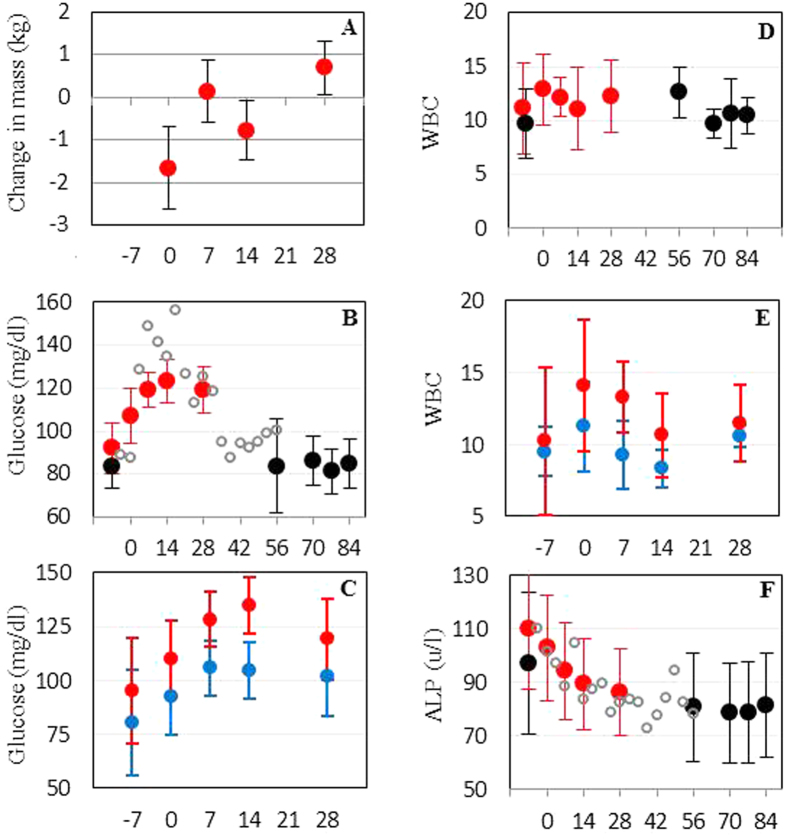
Correlates of acute stress. (**A**) Mean change in body mass between sedations (1 kg equals 1.4% of initial mean body mass). (**B**) Serum glucose and (**D**) WBC for ten chimpanzees in this study (red circles), six chimpanzees from previous Ebola VLP vaccine study^14^ (solid black circles), and 26 newly captive baboons^20^ (open black circles). Glucose in this study and the baboon study peaked on about the same day and exhibited similar baseline levels. Higher peak glucose in baboons is consistent with more severe stress. Chimpanzee WBC in this study peaked on Day 0 then plateaued. Chimpanzee WBC in the Ebola VLP study returned to baseline by Day 56. WBC not available for baboon study. The three orally vaccinated chimpanzees that presented voluntarily for sedation (blue circles) exhibited lower values of (**C**) glucose and (**E**) WBC than the three that did not always present voluntarily (red circles). Voluntary presenters showed significantly lower serum glucose (t test Day 7 p = 0.04, Day 14 p = 0.017) and WBC (Day 7 p = 0.039) than non-voluntary presenters.(**F**) Alkaline phosphatase (ALP) in this chimpanzee study and the baboon study peaked on the first sampling day then decayed at similar rates to baseline concentrations that were, in both cases, 71% of the peak value, suggesting that similar mechanisms down-regulate stress responses in the two species. Baboon values have been normalized to the peak chimpanzee concentration to illustrate this similarity. Error bars in all panels are 95% confidence intervals (i.e. 1.96 standard errors).

The psychological pathologies (e.g. repetitive behavior, severe lethargy, self-harm) often seen in victims of severe trauma, chronic psychological stress or extreme social isolation^15^ were entirely absent in study chimpanzee. On trial Day 0 study chimpanzees did, however, exhibit several well-known correlates of hypothalamic-pituitary-adrenal (HPA) axis activation by acute psychological stress^16^,^17^,^18^, including a small body mass loss relative to Day -8 (1.65 kg, paired t-test p = 0.004, Fig. 3A) and rises in serum glucose (p = 0.025, Fig. 3B) and white blood cell count (p = 0.04, Fig. 3C). The values of these correlates indicate relatively mild levels of stress. For example, a previous study found that 45 days after being taken into captivity wild African green monkeys had lost an average of 42% loss body mass^19^ while peak weight loss was 2% in our study chimpanzees (Fig. 3A). Another study^20^ found that serum glucose peaked at 156 mg/dL 25 days after wild baboons were taken into captivity compared to a peak of 123 mg/dL for our study chimpanzees 21 days after being moved from outdoor group housing to indoor experimental housing in paired cages (Fig. 3B). The serum glucose rise observed in chimpanzees that voluntarily presented for sedation was particularly modest (from 80 mg/dL on Day -8 to 106 mg/dL on Day 14) (Fig. 3E): very similar to the values observed in college students anticipating exams^21^.

**Figure 4:**
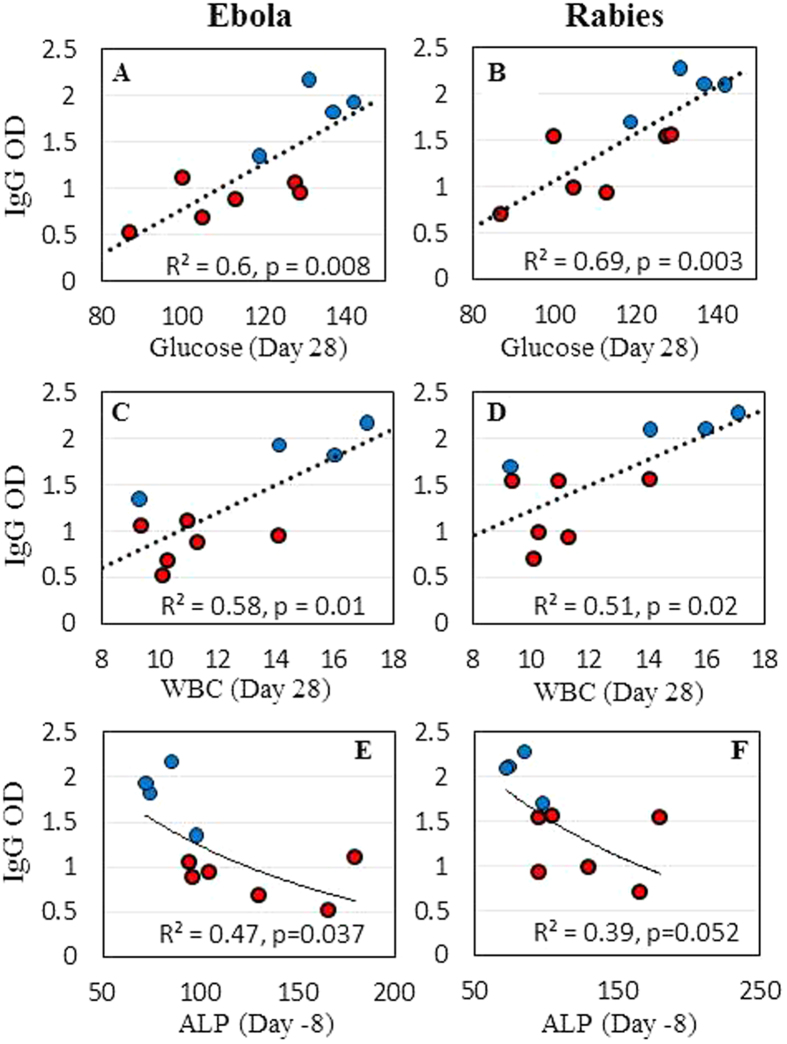
Immune responses enhanced by stress. ELISA optical densities (OD) of EBOV GP-specific IgG (left column) and RABV-specific IgG (right column) were correlated with three well-known correlates of acute stress (**A**,**B**) serum glucose, (**C**,**D**) WBC, and (**E**,**F**) Alkaline phosphatase (ALP). R^2^ and p value in each plot are for univariate least squares regression of each stress correlate versus Day 28 IgG OD at 1:150 dilution. Note that IM vaccinated chimpanzees (blue circles) tended to exhibit higher values for all stress correlates. Because of correlation between stress variables, multivariate stepwise regression was used to test for independent effects of WBC, glucose, and ALP on IgG OD (see main text for results).

Study chimpanzees did not exhibit the immunosuppression often associated with chronic psychological stress^12^. To the contrary, enhanced innate immune activity was indicated by the observed rise in WBC (Fig. 3C). What’s more, humoral immune responses were positively correlated with acute stress responses (Fig. 4). In univariate regressions of Day 28 IgG specific to EBOV GP showed strong positive correlation with Day 28 glucose (least squares regression R^2^ = 0.6, p = 0.008) and Day 28 WBC (R^2^ = 0.57, p = 0.011) and a negative correlation with Day -8 ALP (R^2^ = 0.44, p = 0.036). Day 28 IgG specific to RABV also exhibited positive correlation with Day 28 glucose (R^2^ = 0.69, p = 0.003) and Day 28 WBC (R^2^ = 0.51, p = 0.02) and negative correlation with Day -8 ALP (R^2^ = 0.39, p = 0.052). When stress correlate values for each chimpanzee were averaged across the study, correlations with Day 28 IgG were slightly weaker but still significant in most cases. We tested for independent effects on IgG using stepwise linear regression with a p = 0.05 inclusion threshold. For the single day stress correlate data there was not support for inclusion of more than the best fitting predictor variable in the model (glucose for both Ebola GP IgG and RABV IgG). However, for the data averaged across sampling days the best model for RABV (R^2^ = 0.93) included both glucose (p = 0.015) and WBC (p = 0.01). A model containing WBC (p = 0.011) also came very close to accepting ALP (p = 0.054) as a predictor of EBOV GP. Given the small sample size (10 individuals), the difference between the predictor variables chosen by stepwise regression probably should not be attributed to different functional responses to RABV and EBOV GP but to fairly large variances within three variables whose values are all influenced by the same immune processes. That the model predicting individual averages (which smooth stochastic variance) had stronger inferential power supports this conclusion.

The chimpanzees that showed the strongest signs of stress at Day 28 were also the most stressed before vaccination. Day 28 values of the best univariate predictor of Day 28 EBOV GP IgG, glucose, were strongly correlated with glucose on later days (Day 7 R^2^ = 0.72, p = 0.02; Day 14 R^2^ = 0.78, p = 0.007; Day 28 R^2^ = 0.72, p = 0.02). The correlation between Day 0 and subsequent sampling days was weaker for WBC (Day 7 R^2^ = 0.37, p = 0.06; Day 14 R^2^ = 0.44; p = 0.037; Day 28 R^2^ = 0.15, p = 0.15) but extremely strong for ALP (all days R^2^ > 0.9, p < 0.00001). The fact that high stress values preceded vaccination implies that the correlation between stress and IgG reported above was not just a byproduct of the HPA axis arousal that often follows vaccination with replication competent vaccines. Rather, the causal arrow appears to point in the direction seen in human vaccine trials, from HPA arousal by acute stress to enhanced humoral immune response^12^.

These results raised the possibility that the higher IgG concentration observed in IM chimpanzees were caused by more intense anxiety about sedation rather than the larger inoculum sizes delivered via IM vaccination. To test this possibility we conducted three separate bivariate regressions of Day 28 IgG, pairing each Day 28 stress correlate, S, with a dummy variable representing vaccination mode (IM = 1, oral = 0)


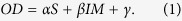


To distribute the stress correlate values on a 0–1 scale comparable to the vaccination treatment we transformed the stress value for each individual, i, as


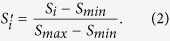


With this rescaling the fitted values of α represent the additional Day 28 IgG produced by increasing from the lowest to highest stress correlate value. For EBOV GP the best model (R^2^ = 0.88) included WBC (α = 0.586, p = 0.048) and IM (β = 0.725, p = 0.004), implying that the effect of WBC on IgG was 81% as large as that produced by IM (versus oral) vaccination. For RABV, glucose (α = 0.89, p = 0.041) produced the best fit (R^2^ = 0.9) and had an effect 82% stronger than IM vaccination (β = 0.49, p = 0.061), although under a strict p = 0.05 stepwise inclusion rule IM would not be included with glucose in the best model. These results imply that stress had an impact on IgG concentration at least as large as mode of vaccination.

## Discussion

Filorab1’s immunogenicity and lack of side-effects in captive chimpanzees bode well for its use to protect wild chimpanzees and gorillas endangered by EBOV. Filorab1 produced immune responses comparable to those observed in the only previous EBOV vaccine trial on captive chimpanzees using a virus-like particle (VLP) vaccine^22^ but did so with only one dose rather than the three doses given in the VLP study. Both robust immune response to a single dose and oral delivery are massive advantages for the field vaccination of wild apes that are difficult to locate in dense forest and fear human approach.

We are already taking further steps towards realizing the potential of oral vaccination as an ape conservation tool, including ongoing tests of both oral bait prototypes on wild apes and methods for quantifying and controlling rates of vaccine bait uptake by both apes and non-target species. Important future steps include heat stabilization of vaccine for longer viability under hot forest conditions, non-invasive assays for vaccine safety and immunogenicity, and field trials on wild apes. Because of the special position that wild apes hold in the public imagination, this work could provide a particularly powerful proof of the more general principle that oral vaccination is a safe and efficient way to protect endangered species against a large and growing pathogen threat. EBOV challenge experiments on macaques might also be used to establish the protective effect of oral delivery of filorab1. However, conservation funds are scarce and the cost of such trials conducting such trials in a BSL4 facility would likely equal or exceed that of all other activities combined.

It seems likely that further safety and immunogenicity trials on captive chimpanzees will not be part of the development program. In principle, research that benefits wild chimpanzee conservation is exempt under the new ESA regulations banning medical research on chimpanzees. In practice, all of the biomedical facilities that held chimpanzees have or are in the process of “retiring” their populations to sanctuaries: sanctuaries which are philosophically opposed to invasive biomedical research. The Pan African Sanctuary Alliance also recently voted to oppose biomedical research on chimpanzees at its member institutions. Biomedical research facilities in developed countries other than the United States no longer hold chimpanzees. And extensive informal outreach suggests that, although zoos have the facilities to conduct safe and rigorous trials and are sympathetic to the conservation objectives, they are unwilling to risk the public backlash that hosting vaccine trials might evoke. This really may be the final vaccine trial on captive chimpanzees: a serious setback for efforts to protect our closest relatives from the pathogens that push them ever closer to extinction in the wild.

This study also has some useful implications for future vaccine trials involving other captive species. First, although our relatively small sample size necessitates caution, the results imply that the psychological stress levels of captive study animals may modulate the antibody response to vaccination. This is not surprising given both published results on the substantial effects of stress on human immune responses and the extensive literature on stress responses of captive animals to experimental procedures and housing conditions. It is, however, surprising that so little attention seems to have been paid to this phenomenon in the design and analysis of vaccine trials involving captive animals. Stress induced variation in immune response is a problem both because it reduces inferential power (by introducing variance unexplained by experimental treatments) and because eventual recipients of the vaccine (often humans) will often not be subject to equally high or persistent stress levels. Thus, more effort may need to be put into mitigating and controlling for animal stress: both experimentally and statistically. A good start would be vaccine trials with larger samples in which sources of stress were experimentally manipulated (e.g. oral vs hypodermic immobilization, vaccination without immobilization). The ban on chimpanzee research effectively precludes such trials as a part of our ape conservation program.

The second implication of our study is that the key to successfully mitigating and controlling for stress is to carefully discriminate between acute and chronic stressors. Much opposition to the use of chimpanzees in biomedical research has rested on the assertion that confinement of chimpanzees in small experimental cages during trials subjects chimpanzees to psychological stress of a severity comparable to that induced by persistent torture^11^. However, the relatively rapid attenuation of stress responses in our study suggests that chimpanzees did not suffer severely from severe, chronic stress due to either confinement in small cages or social isolation. For instance, chimpanzees were no longer losing weight by Day 7 and were gaining weight by Day 28 (Fig. 2A). Similarly, WBC peaked at Day 0 and serum glucose at Day 14. Furthermore, the longer VLP vaccine trial conducted earlier at New Iberia using identical housing and handling protocols showed chimpanzees serum glucose decaying to baseline levels by Day 56. In fact, the combined data from the two chimpanzee studies show a pattern of serum glucose rise and fall very similar to that seen in baboons acclimating to captivity^19^ but with a substantially lower peak implying less severe stress (Fig. 3B). Chimpanzee titers for alkaline phosphatase, an enzyme whose serum concentration responds very quickly to acute stress, peaked at Day -8 and decayed approximately exponentially to near background level by day 28 (Fig. 3D): a pattern also very similar to that seen in the baboon study.

The relatively quick rise and fall seen in the values of stress correlates suggest that homeostatic mechanisms successfully down-regulated the chimpanzee stress responses to experimental conditions. Of particular interest is the observation that chimpanzees that did not always voluntarily present for sedation showed mean glucose and WBC values respectively 11% and 15% higher than chimpanzees that always voluntarily presented (t test glucose p = 0.023, WBC p = 0.033), with glucose and WBC peaks above the lowest daily mean value for non-voluntary presenters that were 69% and 46% higher than voluntary presenters. That voluntary presenters showed an earlier serum glucose peak (Fig. 3B) could be interpreted as more rapid down-regulation of the stress response to sedation. Results from the earlier VLP vaccine trial show that chimpanzee glucose did not rise (Fig. 3B) or weight drop (Fig. 3A) in response to closely-spaced sedations at the end of the study (Days 70, 77, and 84), again consistent with down-regulated stress responses to sedation.

These results suggest that contrary to the claims of animal welfare advocates, housing conditions, per se, may not be a major source of severe stress in trials on captive primates. Apparently, husbandry improvements (e.g. paired cages that allow grooming and other social contact) can successfully minimize social isolation and confinement-related stress of the kind responsible for the behavioral and physiological pathologies that originally galvanized animal welfare advocates to oppose biomedical testing on non-human primates. Rather, the most effective focus of efforts to mitigate stress may be on improved protocols for sedation. For example, sedation stress might be reduced by training of study animals to present voluntarily^15^, distraction tactics^23^, oral sedatives^24^, or newly developed non-invasive assays of antibodies^25^ in secreted and excreted body fluids (e.g. urine, saliva, feces). Data on the behavioural response to sedation as well as hematological and physiological correlates of stress should also be used to statistically control for immune response.

## Methods

### Study Animals

Ten chimpanzees at the University of Louisiana Lafayette’s New Iberia Research Center were randomly assigned to two groups, each having an equal number of males and females. Animals had starting weights of 60.25 kg to 87.09 kg and ranged in age from 17 to 30 y. Research was conducted under protocols approved by the Institutional Animal Care and Use Committee at the University of Louisiana Lafayette in compliance with the regulations of the USDA Animal Welfare Act. The facility where this research was conducted is accredited by the Association for the Assessment and Accreditation of Laboratory Animal Care International and adheres to principles stated in the eighth edition of the *Guide for the Care and Use of Laboratory Animals*^26^.

### Vaccine vector

The vaccine vector BNSP333-coZGP (FILORAB1)^1^ was constructed and recovered as previously described. FILORAB1 grown and titered on Vero cells with a final titer of 1.5 × 10^8^ focus forming units (ffu)/ml.

### Immunizations

A total of 10 Chimpanzees were immunized either intramuscularly (IM) (4 animals) or orally (6 animals) with 1.5 × 10^8^ ffu of FILORAB1.

### Safety Assessments

Chimpanzees were monitored daily and blood was drawn on Days -8, 0, 7, 14, and 28. On blood collection days, chimpanzees were weighed (Supplementary Dataset 1) and examined for general health. Vaccine safety was assessed by monitoring standard hematologic (Supplementary Dataset S2) and blood chemistry (Supplementary Dataset S3) readouts. Hematology analyses were performed using EDTA whole blood on an automated hematology analyzer [Beckman Coulter LH780). Reagents were manufactured by Beckman Coulter and validated for use on the Beckman Coulter LH780 analyzer. All blood chemistry analytes including (Glucose, AST and ALT) were analyzed using serum on an automated chemistry analyzer (Siemens Dimension RXL and/or Siemens Dimension Xpand). Reagents were manufactured by Siemens and validated for use on the Siemen’s Dimension analyzer. All hematology and blood chemistry parameters were reviewed following facility SOPs.

### IgG Responses

We used ELISA to test individual chimpanzee and control sera for the presence of IgG specific to EBOV GP and RABV as described previously^27^. Post-challenge sera from two Rhesus macaques vaccinated with filorab1^14^ were used as controls. Results reported in the main text are for 1:150 dilution of serum IgG.

### RABV Neutralizing Antibodies

Sera were heat inactivated at 56 °C for 30 min. Neutralizing activity was determined using the rapid fluorescent focus inhibition test (RFFIT) assay as described previously^28^.

### EBOV Neutralizing Antibodies

Sera and control sera were heat inactivated at 56 °C for 30 mins. 2-fold dilution of the sera samples starting at 1:5 in serum free medium were prepared in 96 well round bottom plates. 35 pfu of VSV- VSVΔG-ZGP-GFP was added to each for 2 hours at 34 °C and transferred to confluent Vero cells in 96 well plates for another 2 hours at 34 °C. This virus-sera mix was then aspirated from the cells and a 100 μl/well overlay of 0.8% low melting agar in Optimem was added. The plates were placed in a incubator 34 °C for 9 hours and fluorescent plaques counted under a microscope. The dilution factor indicating 50% neutralization was reported as the neutralizing titer.

## Additional Information

**How to cite this article:** Walsh, P. D. *et al*. The Final (Oral Ebola) Vaccine Trial on Captive Chimpanzees? *Sci. Rep.* **7**, 43339; doi: 10.1038/srep43339 (2017).

**Publisher's note:** Springer Nature remains neutral with regard to jurisdictional claims in published maps and institutional affiliations.

## Supplementary Material

Supplementary Dataset S1

Supplementary Dataset S2

Supplementary Dataset S3

## Figures and Tables

**Figure 1:**
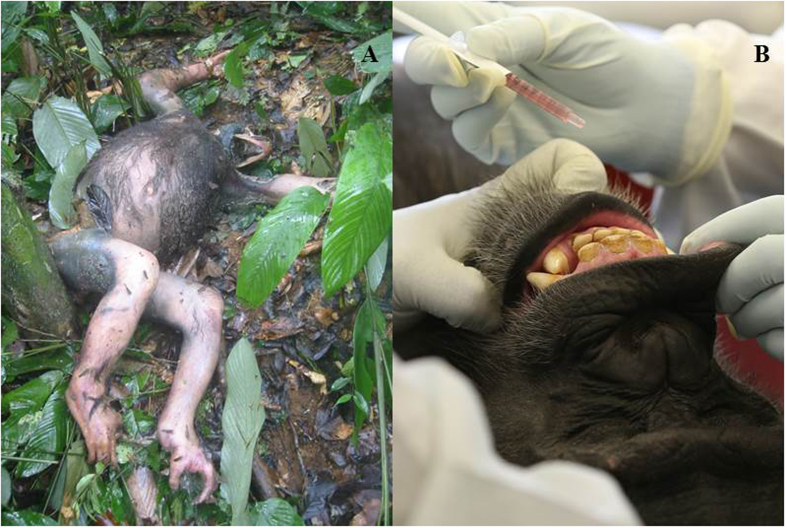
Relative health impact of EBOV infection versus sedation for EBOV vaccination. (**A**) Carcass of a chimpanzee killed by EBOV in Odzala-Kokoua National Park, Republic of Congo. (**B**) Sedated chimpanzee being orally vaccinated against EBOV at the New Iberia Research Center, USA.
